# The inflammatory APRIL (a proliferation-inducing ligand) antagonizes chondroitin sulphate proteoglycans to promote axonal growth and myelination

**DOI:** 10.1093/braincomms/fcae473

**Published:** 2025-02-07

**Authors:** Mashal Claude Ahmed, Tejaswini Kakunuri, Leticia Peris, Delphine Meffre, Elif Nur Yilmaz, Laureen Grewing, Raquel Guerrero González, Benoit Manfroi, Evelyne Gout, Romain R Vivès, Una Fitzgerald, Pascal Schneider, Mehrnaz Jafarian-Tehrani, Tanja Kuhlmann, Bertrand Huard

**Affiliations:** Institute for Advanced Biosciences, University Grenoble-Alpes/INSERM U1209/CNRS UMR5209, La Tronche 38700, France; T-RAIG, TIMC, University Grenoble-Alpes/CNRS UMR5525, La Tronche 38700, France; Institut des Neurosciences, Université Grenoble Alpes, La Tronche 38700, France; UMR-S 1124, University Paris-Cité and INSERM, Paris 75006, France; Institute of Neuropathology, University Hospital Muenster, Muenster 8149, Germany; Institute of Neuropathology, University Hospital Muenster, Muenster 8149, Germany; Institute of Neuropathology, University Hospital Muenster, Muenster 8149, Germany; Institute for Advanced Biosciences, University Grenoble-Alpes/INSERM U1209/CNRS UMR5209, La Tronche 38700, France; CNRS, CEA, IBS, University of Grenoble Alpes, Grenoble 38000, France; CNRS, CEA, IBS, University of Grenoble Alpes, Grenoble 38000, France; Galway Neuroscience Centre, School of Natural Sciences, National University of Ireland, Galway H91 W2TY, Ireland; Department of Immunobiology, University of Lausanne, Epalinges 1066, Switzerland; UMR-S 1124, University Paris-Cité and INSERM, Paris 75006, France; Institute of Neuropathology, University Hospital Muenster, Muenster 8149, Germany; T-RAIG, TIMC, University Grenoble-Alpes/CNRS UMR5525, La Tronche 38700, France

**Keywords:** a proliferation-inducing ligand, chondroitin sulphate proteoglycan, CNS, regeneration

## Abstract

Lesions in the CNS are frequently associated to a detrimental inflammatory reaction. In autoimmune neurodegenerative diseases, a proliferation-inducing ligand (APRIL) produced by CNS-infiltrating inflammatory cells binds to chondroitin sulphate proteoglycans (CSPGs). The latter are well-established obstacles to neural regeneration and remyelination in the CNS by interacting with receptor protein tyrosine phosphatase (RPTP) and Nogo receptor (NgR) families. Here, we are showing that APRIL blocks the interactions of RPTP and NgR with all types of chondroitin sulphate (CS). Functionally, APRIL neutralized the inhibitory effects of CS on mouse and human neuronal process growth. APRIL also blocked the inhibition of CS on mouse and human oligodendrocyte differentiation. Finally, APRIL increased myelination in an *ex vivo* organotypic model of demyelination in the presence of endogenous CSPG upregulation. Our data demonstrate the potential value for a recombinant form of soluble APRIL to achieve repair in the CNS.

## Teaser

Ahmed *et al*. report that a proliferation-inducing ligand secreted in lesions within the CNS is able to counteract the inhibition on neuroregeneration mediated by chondroitin sulphate proteoglycans. This finding renders a proliferation-inducing ligand, a candidate to promote neuroregeneration upon trauma in the CNS.

## Graphical Abstract

**Graphical Abstract:**
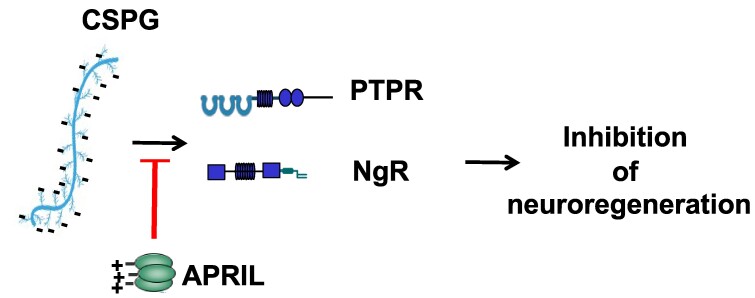

## Introduction

Chondroitin sulphate proteoglycans (CSPGs) contain glycosaminoglycan (GAG) chains of chondroitin sulphate (CS) consisting of repeating disaccharide units made of glucuronic acid and N-acetylgalactosamine. CSPGs come in a variety of types depending on their sulphation pattern on N-acetylgalactosamine and epimerization of the glucuronic acid.^1^ Their functions largely depend on their CS chain types. CS-A and CS-B (also known as dermatan sulphate) harbour a sulphate group on carbon 4 of galactosamine but differ by an epimerization process converting glucuronic acid into iduronic acid in CS-B. CS-E on the other hand harbours disulphated disaccharide units on carbons 4 and 6 of galactosamine. Upon trauma in the CNS, CSPG expression is upregulated to form a glial scar promoting inflammation and inhibiting regeneration.^2-4^ Regarding regeneration, CSPGs act on neurons and oligodendrocytes (OLs) to block axonal growth and migration/differentiation, respectively.^5,6^ CS-A, CS-B and CS-E have all been shown to inhibit axonal growth.^7-9^ The inhibition mediated by CS GAGs depends on their ability to bind to specific receptors from the protein tyrosine phosphatase receptor (PTPR) (PTPRF/PTPRS) and Nogo receptor (NgR1/NgR3) families.^10-12^ Pioneering work using digestion of CS chains by chondroitinase coined the inhibitory function of CS chains on neuronal regeneration.^2,13,14^ Inhibition of CSPG receptor signalling by targeting the associated leucine-rich repeat and immunoglobulin domain-containing Nogo receptor-interacting protein 1 (LINGO-1) was also shown successful preclinically.^15^

A proliferation-inducing ligand (APRIL), also known as the tumour necrosis factor superfamily member 13 (TNFSF13), is a peculiar member in the TNFSF, since it binds to heparan sulphate proteoglycans (HSPGs) in addition to canonical receptors from the TNFRSF.^16^ We recently demonstrated that APRIL also binds to CSPGs upregulated in multiple sclerosis (MS) and neuromyelitis optica (NMO) lesions.^17,18^ In the present study, we analysed functionally the effect of APRIL binding to CSPGs.

## Materials and methods

### PTPRS and NgR1 cloning

Total RNA from mixed glial cultutres was extracted using the RNeasy mini kit (Qiagen). The extracted RNA was treated with DNAse I (Thermo Fisher). RNA was converted into cDNA with Moloney Murine Leukemia Virus SuperScript II Reverse Transcriptase according to the manufacturer’s instructions (Invitrogen). Primers designed for the cloning of soluble mouse PTPRS containing amino acids 1-855 (accession no. XP_006523944) were forward 5′ GC GAATTC GCCACC ATGGCGCCCACCTGGAG 3′ and reverse 5′ CG GTCGAC GCCCTCCTCGCCGTCCAC 3′. Primers designed for the cloning of soluble mouse NgR1 containing amino acids 1-446 (accession no. NP_075358) were 5′ GC GGATCC GCCACC ATGAAGAGGGCGTCCTCC 3′ and reverse 5′ CG GTCGAC ACCCTCTGCGTCCCCTG 3′. We applied 35 cycles on a geneAmp PCR system 2700 from Applied Biosystems. For PTPRS, amplification consisted in initial denaturation at 98°C for 2 min followed by cyclic 98°C for 10 s, 65°C for 30 s and 72°C for 3 min. The Phusion High-Fidelity Polymerase kit (Thermo Fisher) was used along with 4% DMSO in the final reaction mix. For NgR1, amplification consisted in initial denaturation at 94°C for 3 min followed by cyclic 94°C for 30 s, 60°C for 30 s and 68°C for 2 min. The Taq Platinum Polymerase kit (Thermo Fisher) was used. Amplicons were purified using the PCR clean-up gel extraction kit from Macherey-Nagel and cloned with T4 DNA ligase (Promega) into a pCRIII vector encoding for an Fc-tag at the C-terminus of the recombinant proteins.^19^ Due to the disulphide bridge present in the hinge region of the Fc-tag, dimeric forms of NgR1 and RPTPS were produced. Purified plasmids from single colonies using the nucleoSpin plasmid purification kit (Macherey-Nagel) were sequenced (Eurofins Genomics). Single nucleotide mutations were removed with a de-mutation protocol achieved by double PCR as previously described.^20^

### Recombinant proteins

HEK-293T cells (ATCC® CRL-3216) were washed and resuspended in serum-free Opti-MEM culture medium (Gibco). Cells were transfected with the polyethylenimine (PEI, Thermo Fisher) method with a 1:2 DNA/PEI mass ratio. After 6 h, medium was replaced with fresh Opti-MEM for 5 days. Supernatants were then harvested, analysed by western blot after protein separation on a SDS-PAGE and transferred to a PVDF membrane (Bio-Rad). The membrane was blocked with PBS, Tween 20 0.3% and BSA 5% and stained with a donkey α-human IgG conjugated to horse radish peroxidase (HRP) (Jackson ImmunoResearch) followed by clarity western ECL substrate (Bio-Rad). Chemiluminescence was acquired with a ChemiDoc^™^ (Bio-Rad). Recombinant proteins were purified on protein-G-sepharose loaded column (GE Healthcare) following the manufacturer’s protocol and quantified by the Pierce BCA Protein Assay Kit (Thermo Fisher). Fc-mouse EDA-1 and Fc-mouse APRIL_A88_, thereafter referred to as Fc-EDA and Fc-APRIL, respectively, were produced as previously described.^21,22^ Own to the trimeric nature of TNF ligands and the disulphide bridge present in the hinge region of the Fc tag, an hexameric form of APRIL was produced. Thy1-Fc was purchased from Adipogen. Thy-1-Fc and Fc-EDA were used as irrelevant control of Fc-APRIL.

### Enzyme-linked immunosorbent assay

CS of type A from bovine trachea (Sigma), of type B from porcine intestinal mucosa (Sigma) and of type E from squid cartilage (AMS-Bio) were used. CS were biotinylated by coupling the C-terminal carboxyl group of CS to the amine group of biotin hydrazide using 1-ethyl-3-(3-dimethylaminopropylcarbodiimide (Thermo Fisher). Maxisorb 96-well plates (Nunc) were coated with purified Fc-fused recombinant protein at 10 µg/ml. Optimal concentrations of biotinylated CS were determined in preliminary dilution experiments. They were used with or without pre-incubation for 1 h with recombinant Fc-APRIL or Thy1-Fc. Wells were washed twice before the addition of streptavidin-HRP (Thermo Fisher) as the detection reagent, then washed five times before the addition of the substrate 3,3′,5,5'-tetramethylbenzidine (BD Bioscience). The reaction was stopped with sulphuric acid 2N, and optical density was measured at 450 nm using a Victor3 1420 Multilabel Counter (PerkinElmer).

### Cells

Mouse primary neurons and mouse OL progenitor cells (OPCs) were prepared as previously described from E17 prenatal and P6-8 C57/Bl6 mice (Charles River), respectively, with three animals per experiment.^23,24^ Human neural progenitor cells (NPCs) were prepared from induced from pluripotent stem cells (iPSCs) as previously described.^24^ Differentiation of neurons started by seeding NPCs in N2B27 (Gibco) neuronal induction medium containing 1 µM of smoothened agonist (Cayman chemicals), 100 µM ascorbic acid (Sigma), 2 ng/ml of brain-derived neutrophic factor (Peprotech) and 2 ng/ml of glial cell-derived neutrophic factor (Peprotech) with medium replacement every other day. On Day 6, medium was switched to neuronal differentiation by enrichment with 1 ng/ml TGFßIII (Peprotech), 100 µM dbcAMP (Sigma) and 5 ng/ml activin A (Gibco) with the same medium change frequency. Human neurons were used at Day 10. Oligodendrocyte precursor cells were differentiated from NPCs stably transfected with a doxycycline inducible expression cassette containing *SOX10*, *OLIGO2* and *NKX2.6* as previously described.^23^

### Neurite outgrowth assay

Coating of coverslips with CS-GAGs was performed as previously described.^24^ Optimal coating concentrations to avoid cell death were determined in preliminary dilution experiments. Unless stated, control Fc-APRIL and Fc-EDA were used at 15 µg/ml. Mouse and human neurons were incubated for 3 and 1 days, respectively, on coverslips before being washed once in warm PBS and fixed with PFA 4% sucrose 4%. Cells were then washed and permeabilized with PBS Triton X-100 0.2% before being stained with 10 µg/ml of β-tubulin III antibody (clone Tuj1, R&D Systems) diluted in PBS BSA 1% for 1 h at room temperature. Secondary antibody was a goat anti-mouse IgG2a-FITC (Thermo Fisher). Stained slides were mounted in fluoroshield medium containing 4′,6-diamidino-2-phenylindole (DAPI) for nuclear staining (Abcam). Fluorescence microscopy images were visualized with an Axio Imager M2 microscope (Carl Zeiss) with a plan-neofluar ×10/0.75 objective. Pictures were taken with a AxioCam MRc camera. Neurite lengths were measured in a blinded setting using the NeuronJ (https://imagescience.org/meijering/publications/1016/) plugin in ImageJ. The average total neurite length per cell was calculated using 30 cells per conditions.

### Oligodendrocyte progenitor cell maturation assay

Mouse OPCs were detached from the cell surface with trypsin and seeded on coverslips pre-coated with 10 µg/ml of CS-A in 24-well plates. The cells were maintained in proliferation medium for 24 h followed by differentiation OPC Sato medium for 48 h as previously described.^23^ After fixation, cells were stained with anti-mouse platelet-derived growth factor receptor α (PDGFRα) (rabbit, Cell Signaling) and myelin basic protein (MBP) (rat, R&D systems). Human OLs were stained with mouse monoclonal anti-OL marker 4 (O4) antibody (R&D systems) and rat anti-MBP antibody (Abcam). Secondary antibodies were species-specific fluorochrome-conjugated antibodies (Dianova). Stained coverslips were mounted using ROTI mount fluorcare containing DAPI (Dako) and imaged using Zeiss LSM700 confocal microscope. The total number of DAPI^+^ cells was counted using ImageJ. A total of 30 cells were counted manually in a blinded setting for PDGFRα, O4 and MBP expression.

### Organotypic cerebellar slice culture and remyelination assay

Cerebellar slices from one P10 C57/Bl6 mouse (Charles River) were prepared as already described.^25^ Briefly, after demyelination with 0.5 mg/ml lysolecithin (Sigma) in 1 ml of culture medium for 16 h, 5 µg/ml of Fc-APRIL or Thy1-Fc was added. Slices were harvested after 72 h, fixed in 4% PFA for 1 h at RT and stained at 4°C with 1 µg/ml of rabbit polyclonal antibody against calbinding protein (CaBP) (CB38a, Swant) and mouse monoclonal against myelin-associated glycoprotein (MAG) (MAB1567, Merck). Secondary antibodies were species-specific fluorochrome-conjugated antibodies (Thermo Fisher). Slices were also stained for CSPG (mAb CS56, 5 µg/ml, Ams-Bio). Stained slices were mounted in fluoroshield medium with DAPI. Images were acquired and processed using an LSM 510 META confocal microscope. A region of interest was chosen using ImageJ software for each lobule, and a virtual line was drawn transversally to the axonal tracts of Purkinje cells entering in the lobule white matter trench. For quantification of myelination, the percentage of myelinated axons was determined as the ratio of MAG-positive over CaBP-positive fibres crossing the transversal line. For each condition, nine slices were processed from three independent experiments and three lobules were quantified in a blinded setting for each slice.

### Statistics

Analysis was performed using the Prism software (GraphPad Software Inc.). *t* and one-way ANOVA tests were performed for experiments with two and superior to two, respectively, groups. Details on the statistical test are described in the figure legends. A *P*> 0.05 was considered non-significant (ns). Exact *P*-values and *F* ratios for ANOVA tests are indicated.

## Results and discussion

### APRIL binding to CS prevents their interaction with inhibitory receptors

**Figure 1:**
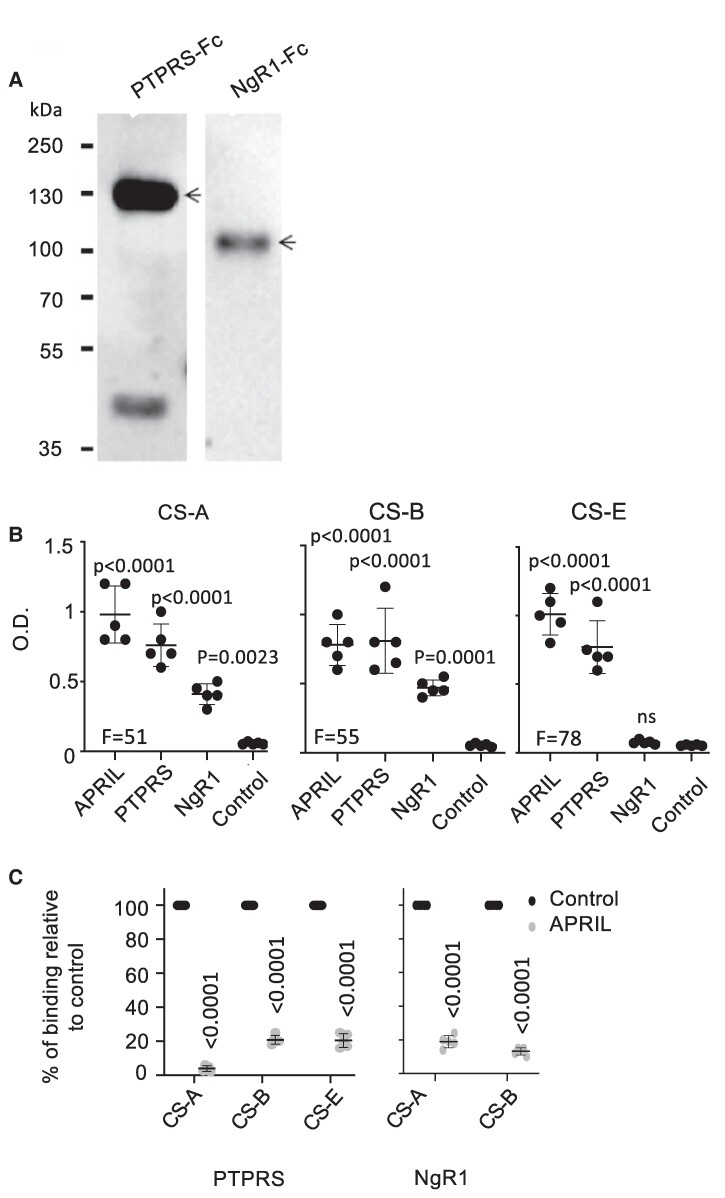
(**A**) Western blot analysis of supernatants from transfected cells with either PTPRS-Fc or NgR1-Fc under reducing conditions is shown. (**B**) Binding of the indicated purified Fc-fused soluble molecules to biotinylated CS was assessed by enzyme-linked immunosorbent assay. (**C**) Binding of recombinant proteins to CS pre-incubated with 10 µg/ml of Fc-APRIL or Thy1-Fc control was assessed by enzyme-linked immunosorbent assay. One hundred per cent was defined by the O.D. obtained with control-treated conditions. CS were used at 50 µg/ml, except CS-E used at 1 µg/ml. Data are presented as mean ± SD of five experiments. Paired one-way ANOVA with Dunnett’s post-test was performed in **B**. A one-sample *t* test was performed in **C**.

We first analysed production of Fc-fused recombinant proteins from inhibitory receptors by Western blot in the supernatant of transfected cells. Under reducing conditions, PTPRS-Fc and NgR1-Fc were detected at their expected sizes of 130 and 100 kDa, respectively, taking into account the presence of three glycosylation sites on each molecule (Fig. 1A). An additional band around 45 kDa containing the Fc fragment and most likely a cleaved fragment of PTPRs was present in the PTPRs-Fc sample. The full uncropped membrane is shown in Supplementary Fig. 1. We then assessed binding of our purified recombinant proteins to different types of CS. For this experiment, we monitored interaction of coated Fc-fused PTPRS and NgR1 to biotinylated CS-GAGs by enzyme-linked immunosorbent assay. Fc-APRIL was used here as a positive control. CS-A, CS-B and CS-E bound to APRIL and PTPRS (Fig. 1B). CS-A and CS-B bound to NgR1. In this assay, pre-incubation of CS with Fc-APRIL inhibited binding to PTPRS and NgR1 by up to 80% (Fig. 1C). Overall, these results demonstrate that APRIL by binding to CS impairs their ability to interact with inhibitory receptors from the PTPR and NgR families.

### APRIL antagonizes neurite outgrowth inhibition by CS

**Figure 2:**
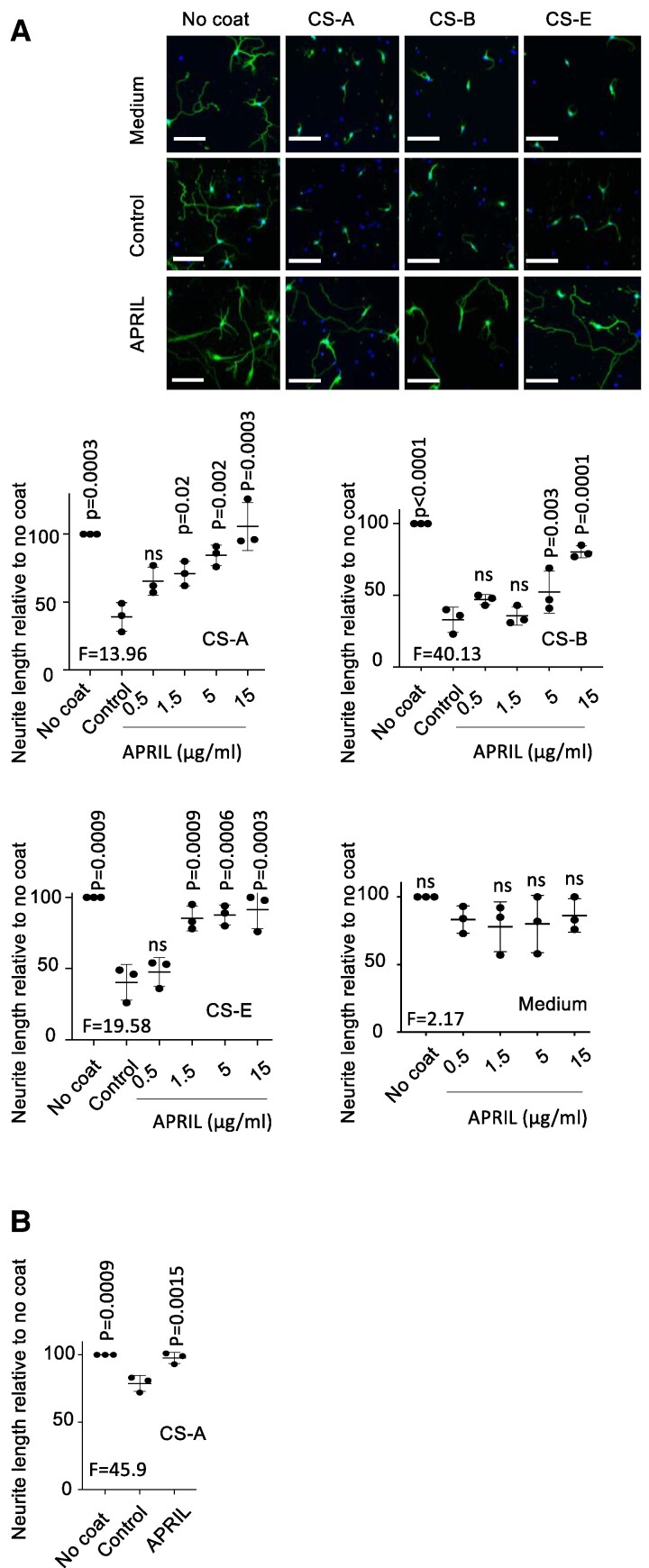
Mouse neurons were cultured for 3 days on either surface coated with chondroitin (CS)-A/CS-B at 10 µg/ml and CS-E at 0.1 µg/ml or non-coated control (no coat) and stained for β-tubulin III (green) and with DAPI (blue). (**A**) Representative images are shown (upper panel). Scale bar = 100 µm. Quantification of total neurite length per cell with the indicated concentrations of APRIL in µg/ml is shown (bottom panel). Control was Thy-Fc. (**B**) Human induced pluripotent stem cell-derived neuronal progenitor cells were grown for 24 h on the indicated substrates. Quantification of neurite length is shown. Control was Fc-EDA. Data are presented as mean ± SD of three independent experiments. Paired one-way ANOVA with Dunnett’s post-test was performed.

We next investigated the effect of APRIL-Fc on neurite growth from neurons in the presence of coated CS-GAGs. Culture of mouse neurons on plates coated with CS-A, CS-B and CS-E inhibited neurite growth by more than 50% (Fig. 2A). We obtained maximal recoveries, close to 100%, with 15 µg/ml of Fc-APRIL but not with control Thy1-Fc. Fc-APRIL did not affect by itself neurite lengths in the absence of CS coating. Having demonstrated that all CS types were impaired in their inhibitory activity on neurite growth by APRIL, we next used CS-A as a representative inhibitory CS as reported by others.^8^ We used human neuronal cells made from iPSC-derived NPCs. The NPC population was visualized by a double staining for the transcription factor Ssox1 and the intermediate filament nestin. NPCs were cultured in differentiation media and were able to grow tubb-3^+^ neurites (Supplementary Fig. 2). CS-A coating also inhibited, although to a lesser extent, neurite growth from human cells (Fig. 2B). We could not achieve a more pronounced inhibition neither with higher concentrations of coated CS-A nor with other CS types. Under these conditions, Fc-APRIL also impaired CS-A inhibition. In conclusion, binding of APRIL to CS resulted in a dose-dependent impairment of their inhibitory activity on both mouse and human neurons.

### APRIL binding to CS allows maturation of OPCs and myelination activity

**Figure 3:**
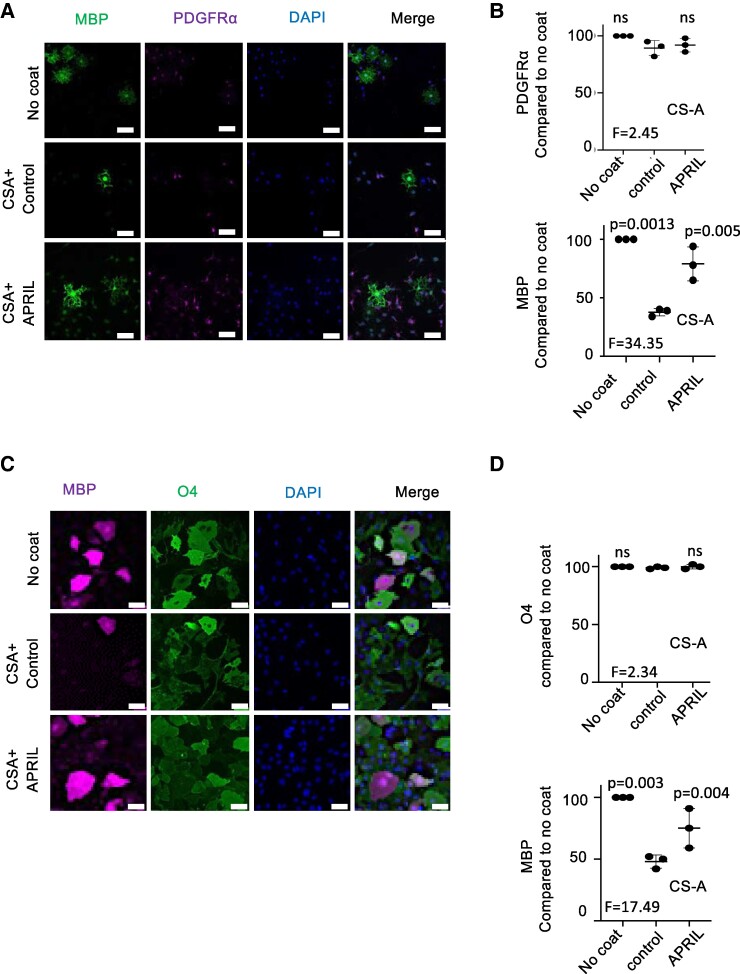
(**A**) Primary mouse OL precursor cells were cultured for 48 h on either CS-A coated or non-coated (control) substrate and stained for MBP, platelet growth-derived factor receptor α (PGDFRα) and with DAPI. Representative images are shown. Scale bar = 50 µm. (**B**) Quantification of the average count of PDGFRα^+^ and MBP^+^ cells among the total number of DAPI^+^ cells compared to control cultures is shown. (**C**) Human induced pluripotent stem cell-derived neuronal progenitor cells were differentiated into OPCs and re-plated onto the indicated substrates. At Day 21, cells were stained for MBP and with DAPI. Representative images of mature OPCs are shown. Scale bar = 50 µm. (**D**) Quantification of the average count of MBP^+^ cells among the total number of DAPI^+^ cells compared to control cultures is shown (right panel). Control for all experiments was Fc-EDA. Data are presented as mean ± SD of three independent experiments, with *n* = 10 fields at ×20 magnification per condition per experiment. Paired one-way ANOVA with Dunnett’s post-test was performed.

**Figure 4:**
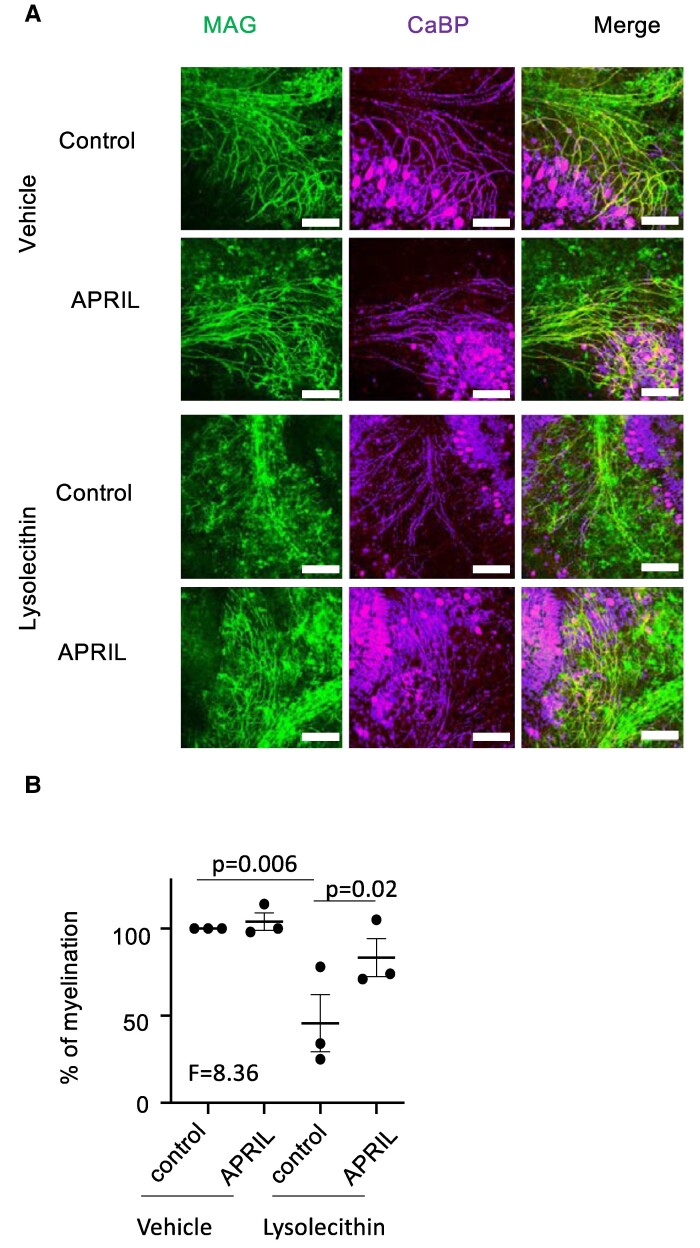
(**A**) Lysolecithin- or vehicle-treated parasagittal slices of mouse cerebellum were cultured with medium containing control Thy1-Fc and Fc-APRIL. Representative immunohistochemistry images of cerebellar lobes stained with MAG for myelin and CaBP for axons at Day 3. Scale bar = 100 µm. (**B**) Quantification of remyelination as a percentage of MAG^+^ CaBP^+^ myelinated axons is shown. Control was Thy-Fc. Data are presented as mean ± SEM of *n* > 18 lobes per condition from three independent experiments. Paired one-way ANOVA with Bonferroni post-test was performed.

We next performed tests on OLs. Coated CS-A did not modulate the proliferation of mouse PDGRα^+^ OL precursor cells (OPCs), while it significantly reduced the number of MBP^+^ mature OLs obtained at the end of the culture (Fig. 3A and B). Pre-incubation of CS-A with APRIL significantly reverted this effect. We then performed this experiment with human OPCs derived from iPSC. Again CS-A coating did not modulate the proliferation of O4^+^ OPCs, but it reduced by approximately 50% the maturation in MBP^+^ OLs (Fig. 3C and D). As for mouse cells, the maturation efficiency was significantly restored in the presence of APRIL. The absence of inhibitory effect for CS-A observed on the proliferation of OPCs is consistent with the report from Sun Y *et al.*^26^ Finally, we tested myelination in the *ex vivo* model of pup-derived cerebellar slice cultures treated with lysolecithin. Myelinated axons were quantified as described in Supplementary Fig. 3. In this model, endogenous CSPG expression increased in response to lysolecithin treatment as previously shown by others^27^ (Supplementary Fig. 4). Under these conditions, Fc-APRIL-treated slices exhibited a greater percentage of myelinated axons at the end of the assay (Fig. 4A and B). Taken together, these experiments show that APRIL is able to counteract CS inhibition on the differentiation of mature OLs and myelination.

We previously showed that injection of recombinant APRIL is able to decrease severity in the mouse model of MS experimental autoimmune encephalitis.^17^ In the present study, we are further showing that APRIL binding to CSPG impairs the ligand functions of the latter by inhibiting their interactions with receptors from the PTPR and NgR families, hence delivering the brake on regeneration in CNS lesions. In multiple sclerosis and optica neuritis, APRIL is produced by innate immune cells infiltrating the CNS, implying that the inflammatory reaction occurring in these diseases may mediate favourable regenerative actions. This is consistent with recent findings arguing that inflammation mediated by innate immune cells may indeed favour remyelination.^28^ Remyelination associated to full regeneration able to diminish patient disability is still an unmet clinical need in neurodegenerative diseases.^29^ Today, CSPG targeting is definitely considered as one emerging therapies to promote regeneration.^30^ According to our study, we propose to add APRIL on the list of molecules to target CSPGs in the CNS.

## Supplementary Material

fcae473_Supplementary_Data

## Data Availability

Data sharing is not applicable to this article as no new data were created or analysed in this study.
