# Supplementary material for: The inflammatory APRIL (a proliferation-inducing ligand) antagonizes chondroitin sulphate proteoglycans to promote axonal growth and myelination
Source: Brain Commun. 2025 Feb 7;7(1):fcae473. doi: 10.1093/braincomms/fcae473 (PMC11803424; doi:10.1093/braincomms/fcae473)
Supplement: fcae473_Supplementary_Data [file fcae473_supplementary_data.pdf]

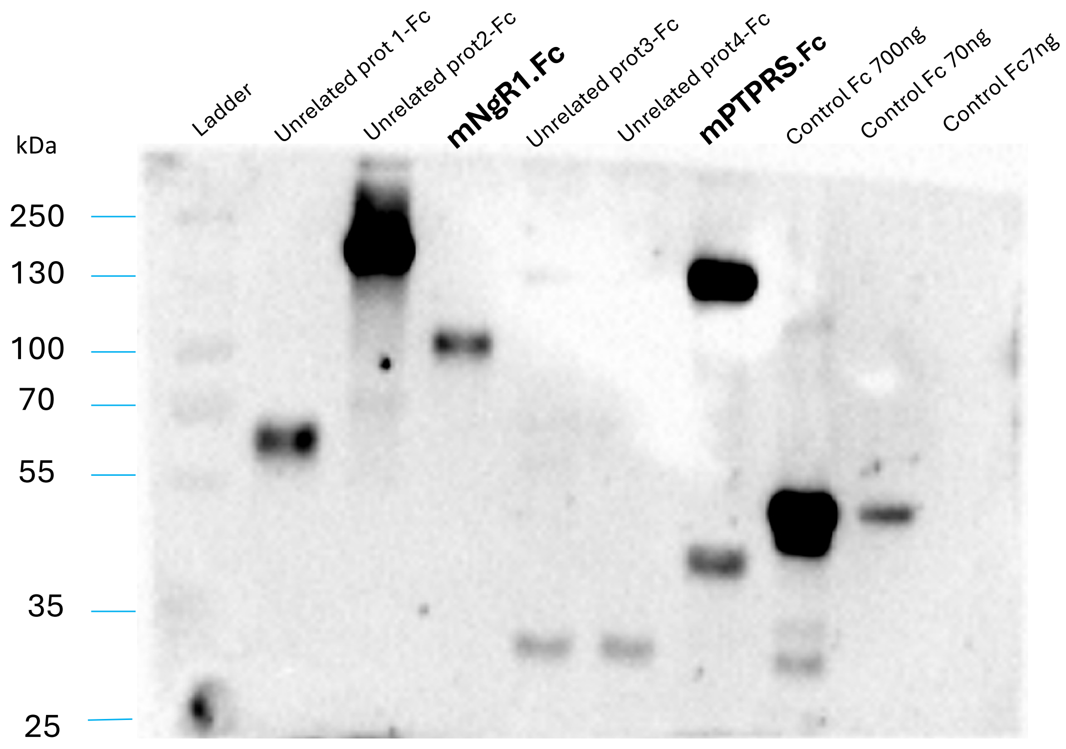

Supplementary figure 1: Uncropped membrane corresponding to figure 1A. Note that Fc-tagged proteins unrelevant to the study was present on the gel. Control Fc was Fc-APRIL.

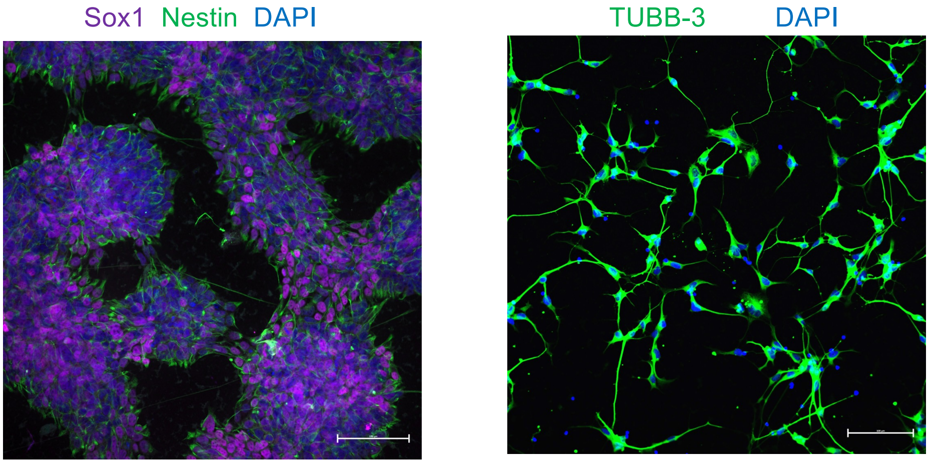

Supplementary figure 2: Differentiation of neural progenitor cells into neurons
Representative images of neural progenitor cells (left panel) stained with Sox1 (red), Nestin (green) and DAPI (blue), scale bar = 100 µm; Neurons (right panel) on the non-coated surface stained for β-tubulin with TUBB-3 (green) and DAPI (blue). Scale bar = 10 µm.

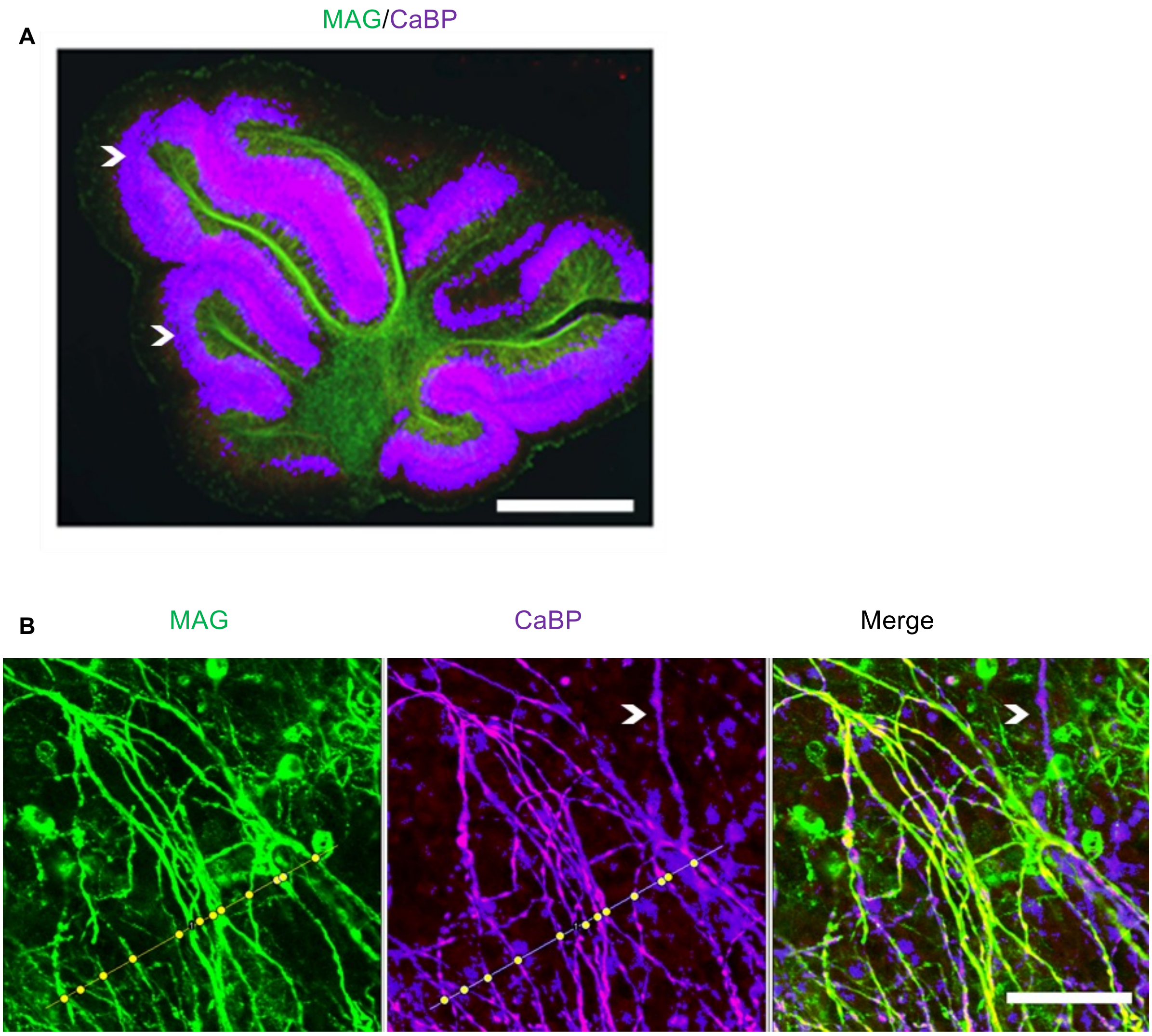

Supplementary figure 3: Quantification of myelination in organotypic cerebellar slices.

A) Low magnification images of a parasagittal cerebellar slice stained with anti-myelin-associated glycoprotein for myelin (MAG, green) and anti-calbinding protein for axons (CaBP, red). Arrows indicate quantified lobes. Scale bar = 0.5 mm. B) High magnification images of cerebellar fibers. The quantification of myelination as a percentage of myelinated axons is defined by the number of MAG-positive fibres over the number of CaBP-positive fibers crossing a virtual line (yellow dots in left and middle panels). An unmyelinated axon is shown (white arrow). Scale bar = 50µm

A

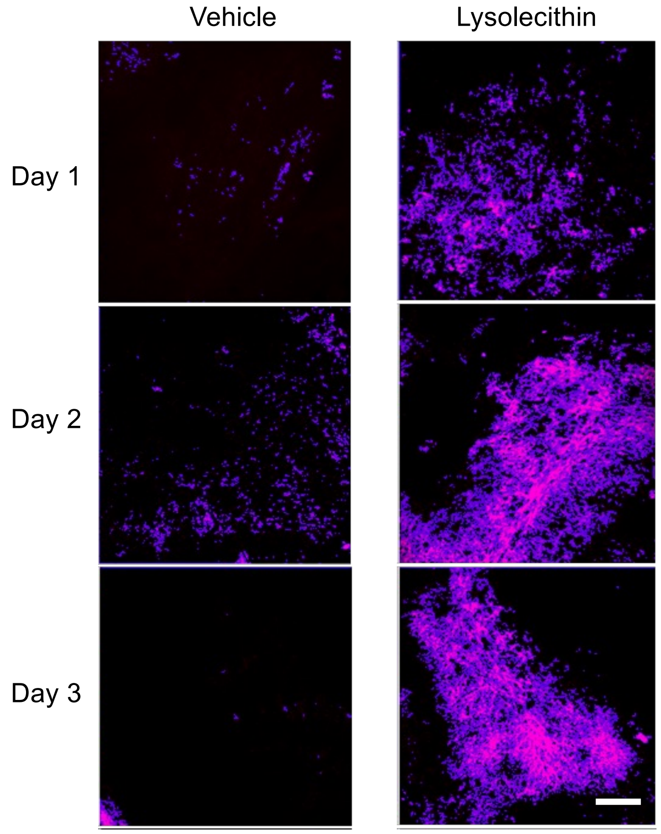

B

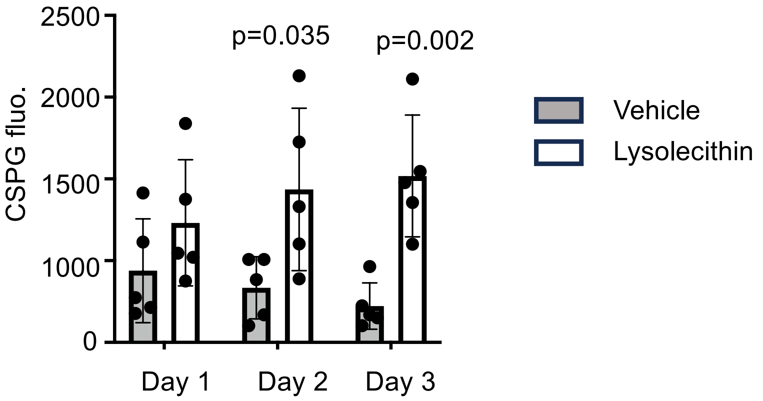

Supplementary figure 4: Upregulation of chondroitin sulfate proteoglycan following cerebellar demyelination.
A) Representative immunohistochemistry images of a cerebellar lobe stained with the anti-CS-56 (red) for CSPGs. Scale bar = 100µm. B) Quantification of chondroitin sulfate proteoglycan (CSPG) expression in cerebellar lobe white matter. Experiments was performed twice with five lobes quantified in total. Each dot represents one lobe. Multiple *t* tests were performed.
